# Fetal Fibronectin and Cervical Length as Predictors of Spontaneous Onset of Labour and Delivery in Term Pregnancies

**DOI:** 10.3390/healthcare10071349

**Published:** 2022-07-20

**Authors:** Delia Grab, Bogdan Doroftei, Mihaela Grigore, Ovidiu Sebastian Nicolaiciuc, Sorana Caterina Anton, Gabriela Simionescu, Radu Maftei, Maria Bolota, Ciprian Ilea, Gabriel Costachescu, Emil Anton

**Affiliations:** 1 Department of Obstetrics and Gynecology, University of Medicine and Pharmacy “Grigore T. Popa”, 700115 Iasi, Romania; delianicolaiciuc@yahoo.com (D.G.); bogdandoroftei@gmail.com (B.D.); mihaela.grigore@umfiasi.ro (M.G.); gabi.ginecologie@gmail.com (G.S.); bolota882003@yahoo.com (M.B.); cilea1979@yahoo.com (C.I.); gcostachescu@gmail.com (G.C.); emil.anton@yahoo.com (E.A.); 2 Clinical Department, Clinical Hospital of Obstetrics and Gynecology “Cuza Voda”, 700038 Iasi, Romania; dr.radu.maftei@gmail.com

**Keywords:** fetal fibronectin, cervical length, term birth, delivery prediction, labour onset

## Abstract

(1) Objective: This study aimed to determine whether qualitative fetal fibronectin and transvaginal sonographic measurement of cervical length are effective in predicting delivery in term pregnancies within 5 days of the test. (2) Methods: We examined 268 women with singleton pregnancies presenting themselves at 37^+0^–40^+4^ weeks (median 38 weeks + 1 day) of gestation with irregular and painful uterine contractions, intact membranes and cervical dilatation less than 2 cm. All women were admitted to hospital up to 72 h after birth. On admission, a qualitative fetal fibronectin test was performed in cervicovaginal secretions and transvaginal sonographic measurement of cervical length was carried out. The primary outcome measure was delivery within 5 days of presentation. RESULTS: Among the women who delivered within 5 days after admission, 65.2% had positive fFN assessment, 43.5% had cervical length below 26 mm, 52.2% had the age > 32.5 years, 34.8% were nulliparous and 56.5% had gestational age ≥ 275 days. Logistic regression analysis demonstrated that significant contributors to the prediction of delivery within 5 days were fibronectin positivity, cervical length ≤ 26 mm, maternal age > 32.5 years and gestational age ≥ 275 days, with no significant contribution from parity. (3) Conclusions: Qualitative fetal fibronectin test and transvaginal cervical length measurement in term pregnancies are useful tests for predicting spontaneous onset of labour within 5 days. It helps women and healthcare providers to determine the optimum time for hospital admission.

## 1. Introduction

The initiation and progress of labour is beyond voluntary control and under the influence of multiple physiological, psychological, and iatrogenic factors. The question concerning the time of delivery is important for the woman and her family as to the obstetrics team, placing a considerable strain on all those mentioned before [1]. Knowing the time of delivery has psychological benefits for the women and is extremely important for the obstetric and administrative team, who can timely assess the management pathways, the opportunity to transfer the patient between units, and ascertain the man-power, space, technical, and economic resources to sustain the event [2]. 

The appraisal of impending labour is commonly done by a combination of clinical symptoms, abdominal pain and uterine contractions, and clinical assessment of cervical dilatation. However, there is no method to accurately predict the time of the onset of labour at term. With a diagnosis of pre-labour, “false” labour, or latent phase of labour, most women will be discharged after assessment, awaiting at home the spontaneous onset of labour, whereas some women will be hospitalized for monitoring under the presumption of impending onset of labour. Consequently, women removed from their communities, particularly when psychological, financial, and transport issues confront the clinical situation, may undergo prolonged hospitalization with increased administrative and economic costs, in addition to heightened maternal stress and morbidity due to extended hospital admissions [3,4,5].

Therefore, ongoing attempts aimed at identifying methods to determine with precision the onset of spontaneous labour and, consequently, the optimum time for hospital admission. To date, the Bishop score is the single established tool used in clinical practice to predict labour, despite concerns regarding the reliability and interobserver variability of the method [6,7]. Thus, there is a need for a prediction tool of labour onset at term, a test that is simple, statistically sound, non-invasive, cost-effective, commercially available, and affordable in low-resource settings. Evolving evidence suggests that fetal fibronectin (fFN) assessment in cervicovaginal secretions and sonographically measured cervical length seems promising in predicting preterm delivery within 7 days [8,9,10]. Fetal fibronectin is a glycoprotein produced by the cells at the uteroplacental interface that has been shown to leak from the choriodecidua through the cervix and into the vagina before the onset of labour [11,12]. Qualitative and quantitative fast-reactive fFN tests have been developed and are used routinely in clinical practice for the prediction of preterm delivery and premature rupture of membranes (PROM) [13,14]. While fFN testing shows an excellent prediction of preterm delivery, its connection to term birth is less clear. After an initial impetus to using fFN to predict term labour and the success of induction of labour at term in the 1990s [15,16,17,18,19], the interest decreased until recently, when newer studies showed a medical and economic benefit [20,21,22]. Transvaginal ultrasound measurement of the cervical length (TVCL) has been reported to be a simple and reproducible examination/marker to predict the time of delivery; however, the results of previous comparative studies are contradictory [23,24,25].

To this end, the objective of this prospective study was to assess the efficacy of fFN test and ultrasound measurement of cervical length (mm) in predicting delivery within 5 days from the test, in women with term pregnancies and pre-labour from north-east Romania.

## 2. Materials and Methods

This was a prospective observational study of sonographic measurement of cervical length and determination of fetal fibronectin positivity in cervicovaginal secretions in women with term pregnancies and pre-labour presenting themselves to the triage of the Obstetrics and Gynecology Hospital Cuza Voda from March 2019 to February 2022. The pre-labour or “false” labour was defined as irregular uterine contractions with or without lower abdominal pain and/or back pain not associated with progressive changes in the cervix dilatation.

The inclusion criteria were: age over 18 years, singleton, cephalic, term, uncomplicated pregnancy, planned for vaginal delivery, and intact membranes. Gestational age was calculated from the menstrual history and by an ultrasound scan in early pregnancy. In the study, we included women with pregnancies between 37^+0^ and 40^+4^ weeks of gestation. Exclusion criteria included: (a) women in active labour, defined as regular, painful uterine contractions resulting in progressive cervical effacement and dilatation of 3 cm or more, (b) women with severe obstetric and medical conditions, (c) planned induction of labour or Cesarean section delivery, (d) fetal anomalies, (e) history of cervical insufficiency, (f) vaginal spotting, (g) ruptured membranes, and (h) history of previous cervical surgery (cone biopsy, large loop excision of the transformation zone (LLETZ)). Those women who had sexual intercourse, vaginal examination, or transvaginal ultrasound within 24 h before presentation.

The eligible women were informed about the purpose of the study and signed an informed consent before participation. This study was conducted in accordance with the Declaration of Helsinki, and approved by the local institutional ethics board (No. 4089/20.02.2019, Gr. T. Popa University of Medicine and Pharmacy and No. 8848/18.09.2018, Obstetrics and Gynecology Hospital Cuza Voda). Upon hospital admission, women underwent a clinical assessment that included a complete history and clinical examination with an assessment of fetal wellbeing. All women underwent hospitalization up to 72 h after birth, according to our maternity protocol.

On admission to hospital, a sterile vaginal speculum examination was performed to assess the cervical changes and confirm the integrity of the membranes. For women who agreed to participate in the study, a specimen of cervicovaginal secretions was collected from the posterior fornix or endocervix, and qualitative detection of fetal fibronectin was performed as described by the manufacturer (JusChek rapid test, Holzel Diagnostika Handels GmbH, Muenster, Germany). Since the test was qualitative, with a positive cut-off value set at 50 ng/mL or greater, the results of the test were recorded as binary, positive and negative. Subsequently, a digital examination was performed by the attending clinicians, and patients with a cervical dilatation of 2 cm or more were excluded from further analysis. After the digital examination, transvaginal sonography was performed and the cervical length was measured by appropriately trained sonographers, from the internal ostium to the external ostium. Three measurements were performed for the cervical length, and the shortest one was taken into consideration for analysis. The results of the fFN tests and TVCL were recorded on an Excel sheet. Demographic and clinical information were extracted from the medical records and recorded onto the same Excel sheet as the results of the fFN tests and TVCL. The study outcome was delivery within 5 days of presentation.

### Statistical Analysis

Statistical analysis was performed using SPSS Software Version 27.0 for Windows (SPSS Inc., Chicago, IL, USA). Continuous variables were presented as mean ± standard deviation and categorical variables as frequencies and percentages. Student’s *t* test was used to compare normally distributed continuous variables and the Mann–Whitney U test was used for variables without normal distribution. In order to identify the cut-off values of continuous variables we used the ROC analysis, by calculating the AUC value and the optimal sensibility and specificity. The Chi-square test and Fisher approximation method were used to compare categorical variables. Univariate analysis was performed for each recorded variable, and variables with *p*-value < 0.01 in univariate analysis were included in multivariate analysis. For adjusted effect we used the logistic binary regression, Forward LR, in 4 steps. The level of statistical significance was defined as *p*-value < 0.05.

## 3. Results

We recruited 268 consecutive pregnant women with abdominal pain, uterine contractions, and gestational age ≥ 37^+0^ weeks gestation. All women had fFN assessment followed by transvaginal sonographic measurement of cervical length at admission. Delivery within 5 days of presentation occurred in 92 (34.3%) cases. Table 1 presents the demographic and clinical characteristics of the women included in the study. The average age was 29.9 ± 5.9 years, with a range between 18 and 43 years old. Most women were living in a rural area (65.7%), were multiparous (71.6%), had previous vaginal deliveries (70.8%) and delivered vaginally in indexed pregnancy (61.2%). The average time between admission and delivery was approximately 7 days.

We investigated the prognostic factors for delivery in 5 days after admission; the most important are the positive fFN assessment and the cervical length; in addition to these, however, there may be other elements that favor delivery within 5 days after admission, possibly cumulated with the first two.

Positive fFN assessment seems to have a definitory role in identifying the spontaneous onset of labour within 5 days: it was obtained in 62.5% such cases, compared with only 4.5% cases among those with spontaneous onset of labour in more than 5 days and has a very high OR (Odds Ratio) coefficient (39.375) (Table 2).

**Figure 1:**
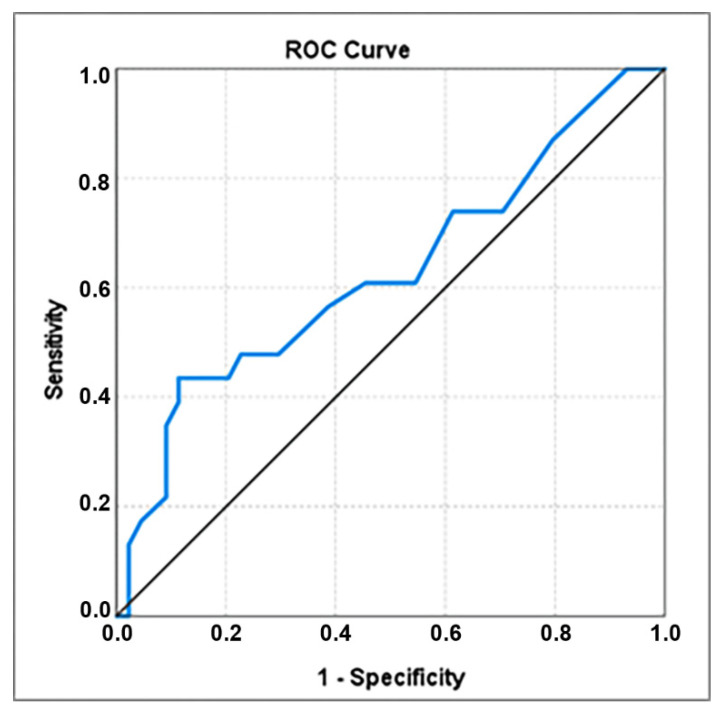
ROC curve for cervical length.

Cervical length at admission was significantly shorter at women who delivered within 5 days after admission (28.83 ± 8.274) compared with the others (32.95 ± 6.371); the ROC analysis revealed the cut-off value of 26 mm, associated with an AUC coefficient of 0.628 (Figure 1) and a good specificity (0.886), useful therefore to identify the real negative cases (Table 3); 43.5% women who delivered within 5 days after admission had the cervical length ≤ 26 mm, a percentage significantly raised compared with only 11.4% women who delivered in more than 5 days (Table 2).

**Figure 2:**
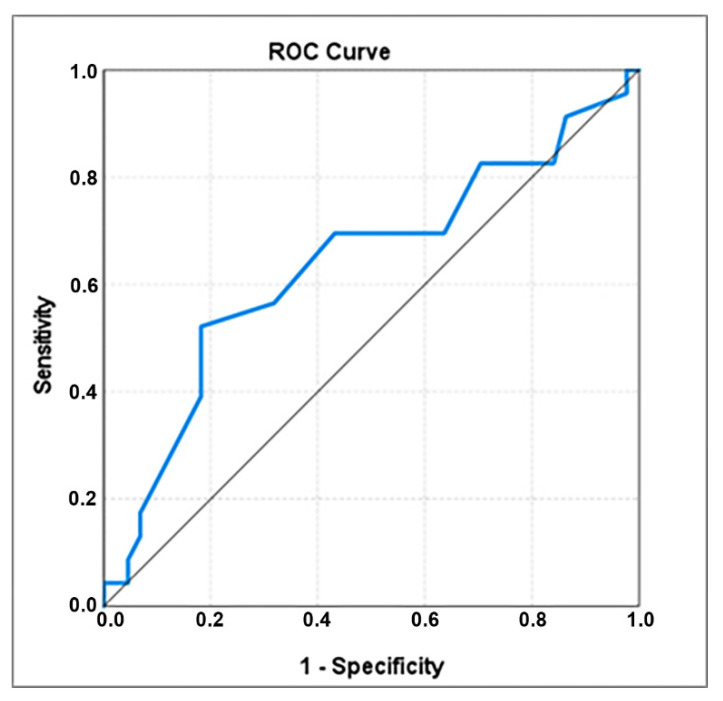
ROC curve for age.

Women who delivered within 5 days after admission were also significantly older than the others, with an average age of 31.48 ± 6247 years and a median of 33 years, compared with 29.14 ± 5491 years and a median of 29.50 years; the ROC analysis revealed the cut-off value of 32.5 years, associated with an AUC coefficient of 0.638 and also a good specificity (0.818) (Table 3, Figure 2). Women older than 32.5 years will deliver within 5 days after admission in 55.2% cases, while the women younger than 32.5 years will deliver in the same time in only 18.2% cases (Table 2). This cut-off value of age has a better discrimination power than the standard threshold of 30 years: 69.6% women who delivered within 5 days after admission had the age > 30 years, compared with 50.0% women who delivered in more than 5 days after admission; even if the recorded difference is also statistically significant, the associated OR is significantly lower (2.286 compared with 4.909 in case of women with age > 32.5 years)—Table 2.

Parity is not statistically significant correlated to delivery within 5 days after admission (Table 2), even if the percentage of nulliparous women is slightly higher among those who delivered within 5 days after admission (34.8%) compared with those who delivered in more than 5 days (25.0%).

**Figure 3:**
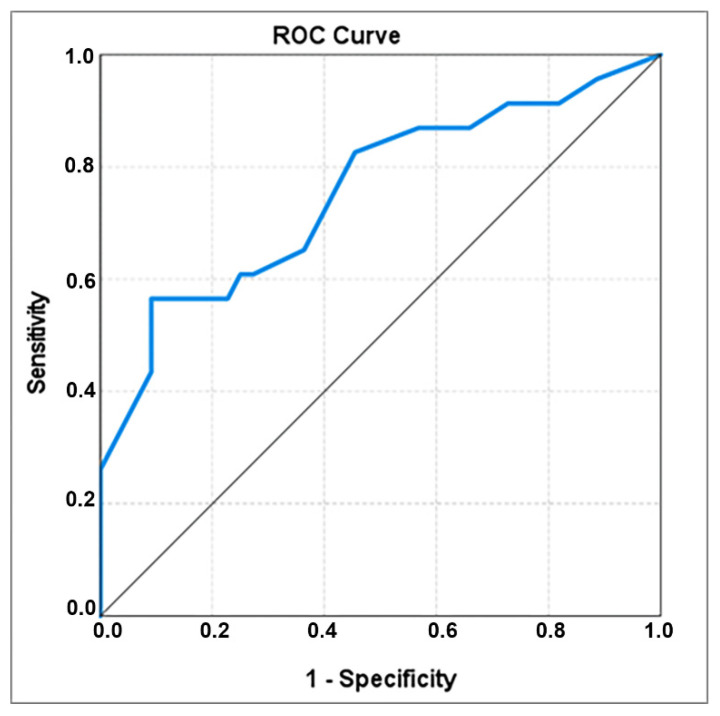
ROC curve for gestational age at admission.

Patients who delivered within 5 days after admission have also had a significantly higher gestational age (273.52 ± 7.754 days) compared with the others (266.64 ± 5.987 days). The optimal cut-off value seems to be of 274.5 days, associated with an AUC coefficient of 0.754 and again a very good specificity (0.909) (Table 3, Figure 3). 56.5% women who delivered within 5 days after admission had the gestational age ≥ 275 days, compared with only 9.1% women who delivered in more than 5 days after admission (Table 2).

Therefore, the prognostic factors for delivery within 5 days after admission identified through univariate analysis were the positive fFN assessment, cervical length at admission ≤ 26 mm, woman’s age > 32 years and gestational age at admission ≥ 275 days. Their multivariate analysis through binary logistic regression shows that all these predictors are important and their combined presence improve the prognostic chances for delivery within 5 days after admission from 65.7% to 88.1%. The probability of delivery within 5 days after admission can be calculated using the equation: Ln (*p*/(1 − *p*) = −6418 + 3910 * (positive fFN assessment) + 1664 * (Age > 32.5) + 1207 * (Cervical length at admission ≤ 26 mm) + 3301 * (Gestational age at admission ≥ 275 days).

## 4. Discussion

The results from this study show that first, the presence of fetal fibronectin in cervicovaginal secretions of women with uncomplicated term pregnancies, respectively a cervical length below 26 mm, can predict the spontaneous onset of labour within a median of five days. Second, a patient with term pregnancy, under the age of 32 or with gestational age more than 275 days is more likely to deliver within 5 days of presentation to the hospital with pre-labour. We showed that qualitative determination of fetal fibronectin and transvaginal sonographic measurement of cervical length are simple tests that can be safely used in almost any environment to timely predict, with good accuracy, the delay until the onset of labour. Used in conjunction with clinical assessment of symptoms, these tests have the potential to allow clinical decisions regarding the optimum time for transfer and admission to hospital and delivery.

The rationale of this study was supported/given by the needs of clinical practice: (i) There is no laboratory or clinical test available up to date to predict with certainty the time of labour onset; (ii) Women with pre-labour or “fake labour” are transferred from their communities to regional centres in the cities, sometimes hundreds of kilometers away, and remain as inpatients for several days, increasing the health care costs and with consequences on the health and economics of women and their families; (iii) These tests are commercially available and successfully used clinically for prediction of preterm birth.

Several previous studies have assessed the effectiveness of fetal fibronectin testing in term pregnancies. In agreement with our findings, a study of 75 pregnant women with term pregnancies from remote areas of Australia, found that the presence of fFN in cervical secretions was associated with increased odds of delivery within 7 days. However, this group found that fFN absence did not reliably exclude the onset of birth [20]. In accord with their observations, we also found that, despite a negative fFN test, some women might have spontaneous onset of labor within the next days; however, this was delayed in rapport to women who had a positive fFN test. Another study by Lockwood et al. showed that low levels of vaginal fetal fibronectin, less than 60 ng/mL, is a predictor of delivery in 95% of postdated pregnancies (≥41 weeks gestation), whereas higher levels of fibronectin predict delivery within 10 days at 39 weeks gestation, suggesting that quantitative determination of fetal fibronectin might be a better test to assess the time of delivery [18]. Mouw et al. also measured quantitatively the fetal fibronectin and found that a concentration of 500 ng/mL in vaginal secretions predicted birth within 3 days [26]. However, in our study, similar to Luton’s study, the threshold for fibronectin detection was 50 ng/mL, suggesting that even low levels of fibronectin can differentiate between the two populations of women with immediate versus delayed onset of labour.

In contrast with our findings, a recent study by Healy et al. found that the presence of fetal fibronectin in cervical secretions did not predict term delivery [27]. However, this research team tested only the negative predictive value of fFN for term delivery, an approach similar to the conventional use of fFN tests for predicting preterm delivery, and did not assess the clinical value of a positive fFN test. Furthermore, the population studied was heterogeneous, including preterm (36 weeks gestation) and term (>37 weeks gestation) pregnancies, the sample size was small (*n* = 17), and the analysis was only descriptive. Taken together, these results suggest that delaying women’s transfer based exclusively on the fFN findings might result in some women birthing in their home communities.

Although TVCL is a reproducible and cost-effective test, studies demonstrate conflicting results regarding its predictive accuracy of term birth.

Our findings suggest that the risk of a patient giving birth in the first 5 days after the measurement is 3.343 times higher if she has a TVCL below 26 mm compared to the opposite situation. Similar to our findings, a study conducted on 199 low-risk antenatal women reported that TVCL measurement between 37 to 40 weeks is a useful test for predicting delivery within 7 days [28]. In this study, the best cut-off of transvaginal cervical length was 2.7 cm for predicting delivery within 7 days. In their studies, Tolaymat et al. and Strobel et al. found that in singleton pregnancies, between 37 and 40, 42 weeks, respectively, TVCL is an independent predictor of the spontaneous onset of labour [29,30]. On the other hand, Meijer Hoogeveen M et al.’s study demonstrated a large inter-individual variation in cervical length before the spontaneous onset of labour at term [31]. Therefore, ongoing studies focus on the role of the association of cervical canal length with other factors, such as fetal fibronectin, in predicting the spontaneous onset of labor at term.

Several observational studies have noted that the combination of fFN and TVCL assessment may improve the prediction of spontaneous preterm birth in women with preterm symptoms. In a prospective study of 665 women with threatened preterm labour, van Baaren et al. found that women with a TVCL ≥ 30 mm or those with a CL 15–30 mm and a negative FFN result were at low risk (defined as 5%) of spontaneous delivery within 7 days [32]. On the other hand, in a multicentre prospective observational study of 195 women presenting with threatened preterm labour, conducted in the United Kingdom and South Africa, delivery within 7 days was more likely to occur in those with short CL than those with a positive fFN test [33].

To our knowledge, this is the first study to interrogate the clinical applicability of combined fFN test and TVCL in clinical decisions regarding hospitalization or transfer of pregnant women at term for labour and delivery. Multiple logistic regression analysis demonstrated that the prediction accuracy for birth within 5 days of hospitalization is 88.1% in the presence of all 4 identified prognostic factors, i.e., positive fFN assessment, cervical length at admission ≤ 26 mm, woman’s age > 32 years and gestational age at admission ≥ 275 days.

## 5. Conclusions

In this study, we found that a risk prediction model including qualitative fetal fibronectin test, transvaginal sonographic cervical length measurement and clinical risk factors showed promising performance in the prediction of spontaneous term delivery within 5 days of the test. Testing fFN in cervicovaginal secretion at term is indicative of spontaneous labour, but its absence does not fully exclude the possibility of the onset of labour.

Further evaluation of the risk prediction model in clinical practice is required to determine whether the risk prediction model improves clinical outcomes if used in practice. Validation of this procedure in accurate prediction of spontaneous onset of labour will not only reduce the duration of hospitalization and lower the expenses in maternities but might also help to avoid the adverse effects of prolonged antepartum hospitalization on the postpartum quality of life.

## Figures and Tables

**Table 1:** Demographic characteristics at baseline.

Demographic Characteristics at Baseline	Total Women (*n* = 268)
Maternal age (years)	29.94 ± 5.857
Age < 30 years	116 (43.3%)
Age ≥ 30 years	152 (56.7%)
Place of living	
Urban	92 (34.3%)
Rural	176 (65.7%)
Parity	
Nulliparous	76 (28.4%)
Multiparous	192 (71.6%)
Gestational age at admission (days)	269.00 ± 7.397
Gestational age at delivery (days)	275.81 ± 6.760
Previous deliveries	192 (71.6%)
Vaginal deliveries	136 (70.8%)
Caesarean section	56 (29.2%)
Mode of delivery	
Vaginal delivery	164 (61.2%)
Caesarean section	104 (38.8%)
Number of days from admission to delivery	6.93 ± 3.689
Cervical length at admission (mm)	31.54 ± 7.334
Delivery within 5 days after admission	92 (34.3%)

**Table 2:** Univariate and multivariate analysis of categorical prognostic factors.

Factor	Delivery within 5 Days after Admission		Univariate Analysis			Multivariate Analysis		
	Yes (*n* = 92)	No (*n* = 176)	OR	95% CI	*p*-Value	OR	95% CI	*p*-Value
Positive fFN assessment at admission	60 (65.2%)	8 (4.5%)	39,375	17,188; 90,202	0.000	49,879	16,896; 147,251	0.000
Cervical length at admission (mm) ≤ 26 mm	40 (43.5%)	20 (11.4%)	6.000	3.222; 11,173	0.000	3.343	1.199; 9.319	0.021
Age > 32.5	48 (52.2%)	32 (18.2%)	4.909	2.803; 8.598	0.000	5.278	2.033; 13.698	0.001
Age > 30.0	64 (69.6%)	88 (50.0%)	2.286	1.341; 3.897	0.002	-	-	-
Nulliparous	32 (34.8%)	44 (25.0%)	-	-	0.092	-	-	-
Gestational age at admission (days) ≥ 275	52 (56.5%)	16 (9.1%)	13.000	6.727; 25.122	0.000	27.126	9.931; 74.097	0.000

**Table 3:** Univariate and ROC analysis of continuous prognostic factors.

Factor	Delivery within 5 Days after Admission		*p*-Value §	Cut-Off Value	Sensibility	Specificity
	Yes (*n* = 92)	No (*n* = 176)				
Cervical length at admission (mm)	28.83 ± 8.274	32.95 ± 6.371	0.001	26	0.435	0.886
Age	31.48 ± 6.247	29.14 ± 5.491	<0.001	32.5	0.552	0.818
Gestational age at admission (days)	273.52 ± 7.754	266.64 ± 5.987	<0.001	274.5	0.565	0.909

§ Mann-Whitney U test.
